# Assessing primary health care provider and organization readiness to address family violence in Alberta, Canada: development of a Delphi consensus readiness tool

**DOI:** 10.1186/s12875-024-02396-3

**Published:** 2024-04-29

**Authors:** Stephanie Montesanti, Anika Sehgal, Lubna Zaeem, Carrie McManus, Suzanne Squires, Peter Silverstone

**Affiliations:** 1 School of Public Health, University of Alberta, Edmonton, AB Canada; 2 Centre for Healthy Communities, School of Public Health, University of Alberta, Edmonton, AB Canada; 3 Cumming School of Medicine, University of Calgary, Calgary, AB Canada; 4 Islamic Family and Social Services Association, Edmonton, AB Canada; 5 Sagesse Domestic Violence Prevention Society, Calgary, AB Canada; 6 Westgrove Clinic, Westview Primary Care Network, Spruce Grove, AB Canada; 7 Department of Psychiatry, Faculty of Medicine and Dentistry, University of Alberta, Edmonton, AB Canada

**Keywords:** Family violence, Intimate partner violence, Modified Delphi technique, Primary healthcare, Readiness, Evaluation, Canada

## Abstract

**Background:**

Family violence, which includes intimate partner abuse, child abuse, and elder abuse, is a serious public health concern. Primary healthcare (PHC) offers a vital opportunity to identify and address family violence, yet barriers prevent the effective implementation of family violence interventions in PHC settings. The purpose of this study is to improve family violence identification and response in Alberta’s PHC settings by exploring readiness factors.

**Methods:**

An integrated knowledge translation approach, combining implementation science and participatory action research, was employed to develop a readiness assessment tool for addressing family violence within PHC settings in Alberta. The research involved three phases: phase 1 involved a rapid evidence assessment, phase 2 engaged a panel of healthcare and family violence experts to explore readiness components in the Alberta context, and phase 3 utilized a 3-round Delphi consensus-building process to refine readiness indicators.

**Results:**

Phase 1 findings from a rapid evidence assessment highlighted five main models/tools for assessing readiness to implement family violence interventions in PHC settings. In phase 2, additional concepts were identified through exploration with healthcare and family violence expert panel members, resulting in a total of 16 concepts for assessing family violence readiness within the Alberta PHC context. The 3-round Delphi consensus-building process in Phase 3 involved nine panelists, who collectively agreed on the inclusion of all concepts and indicators, yielding a total of 60 items for the proposed readiness assessment tool for addressing family violence in PHC within Alberta.

**Conclusion:**

The current study lays the groundwork for future family violence intervention programs, offering insights into key components that promote readiness for implementing comprehensive programs and supporting PHC organizations in effectively addressing family violence.

## Background

Family violence is widely recognized as a serious public health issue [1–3]. It has been defined as behavior directed towards a family member that is physically, sexually, emotionally, psychologically, or economically abusive or coercive and threatening in nature [1, 4–6]. Research on family violence most often focuses on child maltreatment (also known as child abuse and neglect), intimate partner violence (IPV) (also known as spousal violence, dating violence, domestic violence or abuse) and mistreatment of older adults (also known as elder abuse and neglect) [7–12].

While family violence transcends gender and sexual orientation, it is disproportionally perpetrated by men against women and children [13, 14]. Much of the international and national data shows that Indigenous peoples experience higher rates of violence than many other ethnic groups [15, 16]. In Canada, Indigenous women experience 3–11 times the average IPV rate, related to multi-generational trauma and racism experienced by Indigenous communities [17]. Indigenous women are also eight times more likely to be killed by their partners [18]. Violence against Indigenous women is so prevalent in Canada that it has been termed the ‘Missing and Murdered Women, Girls, and Two-Spirited (MMIWG2S +) crisis’, speaking to historical and contemporary realities of assimilationist and genocidal colonial policies and racialized violence [19].

Family violence is also an important issue for health services. The immediate and cumulative effects of violence significantly impact the health and well-being of families and society as a whole [20, 21]. Family violence is associated with long-lasting consequences for physical and mental health in individuals experiencing violence including survivors, perpetrators, and their children [1, 22–27] resulting in the increased use of health services by all patient groups affected by violence [28, 29]. Women exposed to physical violence are shown to have higher mental healthcare utilization compared to women who never had experienced such violence [30]. Thus, the need for an adequate response to this issue by the healthcare sector is clear.

Primary healthcare (PHC) is recognized as a setting uniquely positioned to identify the risk and protective factors for family violence, being an entry point into the healthcare system and the first, or only, point of contact for families with professionals who can facilitate access to specialist care and support [31, 32]. International and national policy guidelines strongly recommend PHC professionals be prioritized for family violence workforce training and service delivery [33–35], because of their historic professional role as having a more holistic and relationship-based approach to healthcare than other professions [36], their interconnections with multiple different community and secondary care services [37], and because family physicians and nurses have more contact with individuals affected by violence and abuse than other health services [38]. Registered nurses/nurse practitioners in PHC practices are often viewed as the most trusted and closest to the patient and their community and can support greatly with the identification and management of family violence [39, 40]. Yet only a minority of women, men, and/or children exposed to family violence are recognized in PHC settings [41, 42]. Despite these mandates, implementation of family violence responses in PHC practices has been slow and incomplete, and little is known about the implementation challenges [43].

Several barriers for implementing family violence responses in PHC have been cited in the literature including a lack of confidence among healthcare providers in both recognizing violence and knowing how to directly raise the topic with patients [44], shortage of time with the patient, lack of system-level supports such as placing screening prompts or reminders [45] for healthcare providers in patients’ electronic health records, cultural barriers (i.e. social acceptability of family violence) [46], strong biomedical approaches, high staff turnover, absence of family violence training or skills, ethnic practices of patients, feeling overwhelmed by the emotional nature of the work or their own experience with violence and abuse, the presence of the patients’ partners [46–48], and limited resources for the implementation of family violence interventions [37, 49–51]. Existing evidence suggests that Indigenous peoples, ethnic minority women, and lesbian, gay, bisexual, trans, asexual, queer, and Two-Spirit (LGBTQ2S) identifying people often avoid seeking support from healthcare providers because of feelings of mistrust and fear toward service providers [27, 52, 53]. This is concerning, given the vital role healthcare providers have in responding to family violence and the poor health outcomes associated with violence.

A comprehensive systems approach to service delivery alongside practice guidelines has been advocated to support a program of sustainable family violence identification and intervention within healthcare settings [54, 55]. Facilitators to identification have also been cited and can include the availability of information on the risks for family violence, local family violence services, screening tools, skills training, and support from a multidisciplinary team [56–58]. Despite a wealth of studies exploring the barriers and facilitators to identification and response to family violence in healthcare settings, much less is known about healthcare providers’ capacity to identify and respond to family violence in PHC settings specifically, and the best ways to support their readiness to undertake the complex work of addressing family violence [54].

### Operationalizing ‘readiness’ for the identification and response to family violence in PHC

The concept of ‘*readiness’* is multi-faceted and has been described as a positive force that may motivate people to make positive changes [50, 59] and their capability to do so [60]. Weiner et al., conceptualized readiness for change at the individual and organizational levels [61]. Change at both levels is intricately linked because the organizational level is a function of change at the level of the individuals that belong to the organization, but also at the level of the organization itself as a collective of individuals. The degree to which organizations are ready for change to adopt new health services or programs can be important predictors of whether an intervention is successfully implemented. Assessing organizational readiness for change involves identifying organizational dynamics, climate, and culture, change processes and individual organizational member characteristics [62]. According to Weiner et al., an organization’s readiness relies on organization members’ motivation to change (change commitment) and belief in their own capacity to change (change efficacy) [61].

Assessing readiness in the context of PHC providers responding to family violence was first used in a scale measuring doctors’ readiness to manage intimate partner abuse [63]. However, in that study, no conceptual definitions of readiness were provided and perceived ‘preparedness’ emerged as a subscale. Across the literature, there are no agreed theoretical foundations for defining what constitutes ‘readiness’ or ‘preparedness’ among healthcare providers to adopt a new family violence model of care, resulting in some conceptual ambiguities. Indeed, some studies have used the term ‘*preparedness*’ [64, 65], but without a clear conceptual framework. It remains unknown if ‘readiness’ and ‘preparedness’ are mutually inclusive in content properties, or whether they are distinct constructs in the context of physicians’ responses to family violence. Most of the existing tools measuring general practitioners’ identification and responses to intimate partner abuse have been informed by the knowledge, attitudes, beliefs, and self-reported behaviours (KABB) framework [66]. Contrarily, in a qualitative study by Po-Yan Leung, interviews with 19 general practitioners described doctors’ readiness to be closely reflected in their emotional response to intimate partner abuse and their motivational beliefs and values that intimate partner abuse is a violation of human rights and doctors have legitimate roles to intervene in cases [67]. Preparedness, on the other hand, was defined by participants in Po-Yan Leung’s study as having consultation plans, the knowledge, resources, and skills to deal with intimate partner abuse. Po-Yan Leung and colleagues [68] led the development and validation of the General Practitioners’ Perceived Readiness to identify and respond to Intimate Partner Abuse Scale (GRIPS), a 30-item scale to measure general practitioners’ self-efficacy, motivational and emotional readiness to address intimate partner abuse. Hegarty et al., described preparedness as a *facilitator* for readiness through increasing knowledge and skills of healthcare providers [50]. The authors identified the following themes for enhancing readiness of healthcare providers to address domestic violence and abuse (DVA): having a personal commitment to addressing DVA, adopting an advocacy approach by working with their patients on pathways to safety and wellness, trusting the relationship between the provider and patient, collaborating with a team, being supported by the health system through training to address DVA, making asking about DVA routine, and allowing time to do the sensitive work with patients. These themes were incorporated into a health practitioners’ readiness framework called the CATCH Model.

Consequently, tools for measuring ‘readiness’ have failed to adopt a systems approach that encompasses structural capabilities beyond providers’ preparedness to include policy, organizational leadership and governance issues, and patient and community awareness and engagement. To support a PHC system’s response to family violence, the *Primary Health Care Family Violence Responsiveness Evaluation Tool* was developed in 2012 to guide the implementation of family violence intervention programs within New Zealand PHC, supporting clinicians in the identification, assessment, and appropriate referral of individuals at-risk or experiencing family violence and allowing for focused program development and quality improvement efforts [33]. This is the only tool designed to provide guidance for PHC on evaluating their organizational context, workplace culture, collaboration with other sectors, and capacity in terms of education and training on cultural safety, to support their organization with the development and implementation of PHC family violence intervention programs. The tool includes indicators for cultural responsiveness to reflect the needs of Māori communities in New Zealand. Consequently, greater understanding is also needed of the factors that shape PHC organization/system readiness to implement or adopt family violence interventions that facilitate approaches to the identification, assessment, and care delivery for individuals and their families experiencing or at risk of violence and abuse.

### Study context

This study was carried out in the Canadian western province of Alberta. Among Canadian provinces, Alberta has consistently had the 3rd or 4th highest rate of domestic violence reported to police among the provinces [4] and the second-highest rate of self-reported spousal violence in the country [4, 69]. Between 2011 and 2020, there were 165 domestic violence deaths in Alberta: 62% female and 38% male [70]. In comparison, the rate of police-reported family violence in Canada was 336 per 100,000 population [71]. However, the true scale of family violence in Canada is unknown because incidents are severely under-reported. Despite the high prevalence of family violence in Canada, the impacts of violence on health and well-being are not well recognized within health policy or practice, meaning health professionals are often responding to this complex problem with limited support.

Community-based anti-violence services in Alberta are delivered by community-based agencies, sexual assault centres, health centres, women’s shelters, and settlement services, with some victim services provided through court-based programs and system-based services. These services are especially important given the specialized knowledge and training of anti-violence workers in interpersonal violence and trauma, and their unique role in supporting individuals in navigating multiple complex systems and processes, including immigration, criminal justice, housing, social services, and healthcare. Alberta Health Services, the province’s regional health authority, funds the Domestic Abuse Response Team, or ‘DART’ program, which offers specialized support to patients of all genders who disclose experience of domestic violence in the emergency department. After assessments, DART staff identify the most suitable next steps for patients, with follow-up occurring three and six months later [72]. The original DART program began at the Red Deer Regional Hospital and is still operational. This program was expanded to twenty-three communities as a result of a COVID-19 grant from the Government of Alberta. However, the DART program ended at all the expansion hospital sites in Alberta as of November 30, 2023, following the conclusion of grant funding. Within PHC practices, providers have primarily responded to violence and abuse through crisis intervention, which includes treatment for those already affected, and identifying situations where violence and abuse is occurring (e.g., early intervention through screening). However, this is not consistently practiced across PHC settings in the province and there is no comprehensive health systems approach to supporting sustainable and equity-oriented family violence prevention and response within PHC.

### Study aims

The overall aim of this study is to support PHC practices in Alberta in developing a systems approach to family violence prevention, identification, and response, and contribute knowledge needed to provide an effective standardized response to family violence within PHC practices. Our primary objectives were: (1) to examine promising interventions for family violence identification and response for implementation in PHC settings; (2) to identify factors at the organization and provider levels which promote or challenge the development of a response to family violence identification and response within PHC; and (3) to engage PHC and anti-violence experts from the province of Alberta in the development of a PHC family violence readiness tool unique to the Alberta context.

## Methods

**Fig. 1:**
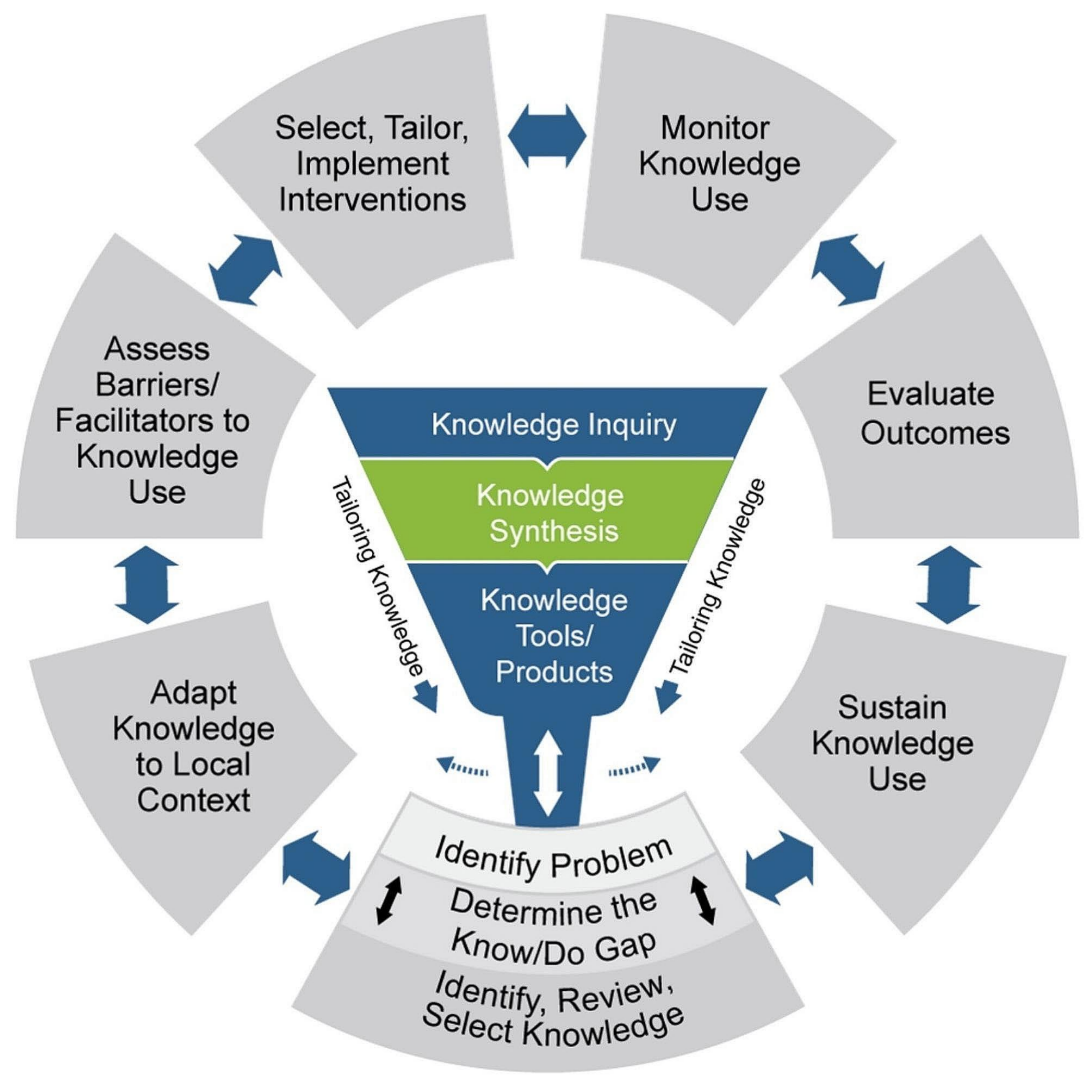
Knowledge to action (KTA) framework

Our study design was grounded in an integrated knowledge translation approach, which is a model of collaborative research, where researchers work with knowledge users who identify a problem and make sure the knowledge is both relevant and applicable for the specific context [73]. We also drew on implementation science research [74, 75] and participatory action research approaches [76] to guide consensus-making on indicators for measuring PHC provider and organization readiness to address family violence. Members of our research team included experts in the field of family violence across health and social service sector and PHC researchers and clinicians from Alberta to generate and build practice-based knowledge. We applied a Knowledge-to-Action (KTA) framework conceptualized by Strauss et al., to examine the iterative and dynamic process of knowledge translation in real-world practice settings that may influence the identification and response to family violence within PHC, centred on the priorities of specific population groups and contexts [77]. The KTA framework comprises steps to support knowledge creation and knowledge application (see Fig. 1). Knowledge creation represents a process in which knowledge is refined, distilled, and tailored to the needs of practitioners, and knowledge application (action cycle), which is the main interest of this study, includes identifying the problem, adapting knowledge to local context, assessing barriers and facilitators to knowledge use, selecting, and implementing interventions, and sustaining knowledge use. The project involved 3 phases to develop the readiness to assess family violence in PHC tool which are described below.

### Phase 1: Rapid evidence assessment

In Phase 1, we conducted a rapid evidence assessment (REA) of empirical studies on family violence interventions in PHC [78]. The assessment aimed to evaluate comprehensive family violence interventions that promote assessment, prevention, and response within diverse PHC settings, while also exploring the key factors that shape PHC provider and systems readiness. The assessment was led by the first author of this article, who supervised a team of research assistants. To identify relevant literature on family violence within PHC settings, we conducted searches on PROSPERO, OVID Medline, EBSCO CINAHL, and SCOPUS, using a controlled vocabulary (such as MeSH terms) and specific keywords related to the concepts of “domestic violence,” “primary care,” “primary health care,” “organizational structure,” “intersectoral cooperation,” and “readiness.” The search was limited to articles published in English between 2005 and 2022. A total of 50 articles were included in this review across a range of study types including systematic reviews, randomized control trials, cohort studies, cross-sectional studies, case studies, qualitative studies, case-control studies, and mixed methods studies. Data from the REA were systematically extracted using a standardized format. This format documented various aspects, including the type of family violence intervention, components of a comprehensive family violence program including training and education of PHC providers and processes for referral to specialized family violence services and supports, evaluation of the family violence program, the role of the PHC team, equity considerations, and any challenges or barriers encountered in implementing family violence programs in PHC.

### Phase 2: exploration of readiness to assess family violence in PHC settings within the Alberta context

In Phase 2, the research team extracted the fundamental components of each family violence intervention to support provider and organization readiness identified in the REA. Additionally, constructs from readiness assessment models/tools used to guide the implementation of family violence interventions in PHC were also extracted. These intervention components and constructs on readiness were compiled and subsequently presented to a panel of experts in the field specifically chosen to offer insights tailored to the Alberta landscape. Panel members were identified by the research team using stakeholder mapping techniques and social network analysis [79–81]. Experts were identified in accordance with the WHO’s recommendation for a holistic, integrated and coordinated response to violence across different sectors and professional disciplines [82]. Recognizing the intersecting determinants of family violence, we aspired to bring together expertise across health and social systems [83]. Additionally, findings from our REA [78] on family violence interventions in PHC demonstrated integrated models that bridge the connection between PHC and specialty care and multi-sectoral collaboration. A key element of the knowledge creation component was the dissemination of an evidence brief to expert panel members synthesizing the findings from the REA. The panel was comprised of family violence experts within the health and social service sector bringing diverse backgrounds and expertise to the table, including a retired sexual assault/forensic nurse with experience in emergency care, sexual assault nurse examiner, domestic violence intensive case manager, psychologist working in a community-based PHC clinic dedicated to immigrants and refugees, family physician working in a mainstream PHC clinic, family counsellor working in an Indigenous-focused PHC clinic, and community agency leaders (see Table 1). In March 2023, an exploratory meeting was organized to assemble the panel of experts. The purpose of this gathering was to present the study’s objectives, engage in discussions regarding the applicability of the identified core components from the REA within PHC settings in Alberta, and explore the need for any supplementary components to be incorporated into an Alberta-specific PHC family violence readiness tool. Further, we followed the steps from the knowledge implementation (“Action Cycle”) component of the KTA framework to understand the problem of family violence within Alberta and adapt the research knowledge to the local context.

**Table 1:** Participants on the expert panel for the Delphi consensus process

Professional role	Sector/Organization type	City	Ethnic or cultural identity	Gender
Retired forensic/sexual assault nurse, and Domestic Violence Program Coordinator	Healthcare	Calgary	European / White	Female
Family Violence Specialist	Social Service Agency	Red Deer	European / White	Female
Sexual Assault and Domestic Violence Program Coordinator	Healthcare (acute care and public health)	Red Deer	European / White	Female
Clinic Director and Registered Psychologist	Social Service Agency (serving immigrants and refugees)	Edmonton	Muslim	Female
Family Physician	Healthcare (primary care)	Spruce Grove	European / White	Female
Family Violence Counsellor	Healthcare (Indigenous mental health)	Calgary	Indigenous	Female
Senior Manager	Social Service Agency serving Indigenous children, youth, and families	Edmonton	Indigenous	Male
Director	Community, Non-Profit Agency	Calgary	European / White	Female

### Phase 3: 3-round Delphi consensus-building process

The Delphi technique uses a series of questionnaires or ‘rounds’ to gather information which are continued until group consensus is reached [84]. We chose the Delphi method because it is an appropriate method for topics where there is limited evidence and wide opinion across professional boundaries. The method is also suitable for when participants work in diverse geographic locations and where there is a need to ensure that individual opinion does not dominate the process of seeking consensus across a wide group of experts. This Delphi method has been successfully used for priority setting in healthcare for vulnerable populations [85–87]. It is also a culturally acceptable method of gaining consensus and has been used in other areas of Indigenous health research [88]. Our modified Delphi process was applied in a participatory action research framework. Like participatory action research projects, the Delphi method produces information that can be put into practice by participants, making it particularly useful for practitioners, organizational leaders, policy, and decision-makers. Throughout the process, we ensured the involvement and ‘interactive dissemination’ [89] of knowledge among experts throughout the project, and the applicability of the results for those involved in the process. Moreover, the relational nature of the Delphi method [90] is also compatible with an Indigenous Relational ontology that emphasizes aspects such as reciprocity, egalitarianism and respect [91, 92].

Scholars have stated that there is no defined agreement on what constitutes the optimum panel size in the Delphi method [93, 94]. Some scholars have recommended Delphi panel sizes to be within the range of 8 to 20 participants [95–97]. Delphi sample sizes depend more on group dynamics in reaching consensus than their statistical power [98]. Nasa, Jain, and Juneja (2021) state that the appropriate size of Delphi panel depends on “the complexity of the problem, homogeneity (or heterogeneity) of the panel, and availability of the resources.” [99]. In accordance with guidance in the research literature on the Delphi method and other important considerations including, the one-year duration of the study, the potential for low response rates that may result from large groups, and the number of Delphi rounds, we deemed 10–12 participants to be sufficient to enable group consensus [100–102]. A sample of 12 experts were invited via email to participate in the Delphi process, and 11 accepted the invitation to join the panel. One participant had dropped out before the first round and another participant did not respond to round 2. To complete the Delphi process, participants were required to respond across all three rounds. Therefore, those who did not respond to round 2 were not invited to participate in round 3. This resulted in a total of 9 panel members who participated in all 3 rounds.

Panelists in the Delphi consensus-building process were gathered to provide a review of the project and its purpose, and they were briefed on the additional concepts that were added from the previous exploratory meeting. To facilitate communication, this introductory session took place online, allowing for an in-depth discussion and ensuring that all panelists had a clear understanding of the Delphi voting process. After the introductory meeting, an online survey for round 1 was shared with all panelists which included voting on the identified concepts, initiating the iterative Delphi process. This first round asked panelists “*how important is this concept to include in the readiness to assess* family violence *in PHC settings within Alberta tool?”* panelists voted on a Likert scale of 1–9 where 1 = not important at all and 9 = very important. Open text boxes were also provided for panelists to share their response rationale and/or any additional feedback. After round 1, a report summarizing the findings was shared with all panelists.

In round 2, panelists reviewed the round 1 results and voted on indicators within each concept. These indicators were extracted from existing tools/models and were adjusted by the research team to reflect the input from panelists in the previous exploratory meeting (see Supplementary materials for a full breakdown of indicators and their sources). This second round asked, “*how important is this indicator to include in the readiness to assess* family violence *in PHC settings within Alberta tool?*” Once again, panelists voted on a Likert scale of 1–9 where 1 = not important at all and 9 = very important. Open text boxes were also provided for panelists to share their response rationale and/or any additional feedback. After this second round, the findings were compiled into a report and circulated to all panelists. The survey was re-administered for the final vote (round 3) using the same question for each indicator as described for round 2.

In order for an indicator to be included in the final set, a consensus was sought through a median vote ranging from 7 to 9, with the condition that there was no disagreement among the panel members. Disagreement was determined using the widely recognized RAND/University of California Los Angeles Appropriateness Method [103], specifically, if the inter-percentile range for a specific question exceeded the inter-percentile range adjusted for symmetry, it was considered indicative of disagreement among the experts.

### Ethics approval

All participants on the Delphi panel provided written informed consent to participate in the study and the study was approved by the University of Alberta Research Ethics Board (Pro00119214).

## Results

### Phase 1: Rapid evidence assessment

Key findings from the REA included the identification of five main models/tools that are used to assess readiness to implement family violence interventions for the identification, assessment, and response to family violence, which are outlined in Table 2.

**Table 2:** Key family violence models/tools identified from REA

Model/Tool	Description
*Physician Readiness to Manage Intimate Partner Violence Survey (PREMIS)*	• 67-item questionnaire • Purpose is two-fold: o To assess the preparedness of physicians to manage IPV o To evaluate the effectiveness of physician IPV education and training Explores providers’ [1] perceived knowledge [2], actual knowledge [3], preparedness, and [4] practice issues surrounding IPV
*Commitment, Advocacy, Trust, Collaboration, Health system support (CATCH) model*	• Developed after identifying five themes that emerged from a meta-synthesis: 1) Having commitment 2) Adopting an advocacy approach 3) Trusting the relationship 4) Collaborating with a team 5) Being supported by the health system Emphasizing these themes may help providers tailor strategies aimed at addressing domestic violence
*Health System Readiness Assessment Tool*	• Identifies health system obstacles and highlights changes needed to successfully implement interventions to address domestic violence. • Composed of seven key health system dimensions: 1) Values (of health policymakers and healthcare staff) 2) Leadership and governance 3) Financing and other resources 4) Coordination and community engagement 5) Health workforce 6) Infrastructure and supplies • Information (related to data collection)
*Healthcare Can Change from Within (HCCW) model*	• Aims to bring about change at multiple levels of the healthcare system, including: a) Clinical staff (change knowledge, skills attitudes, and behavior); b) Clinical environment (change policies, procedures, and workflows; improve patient education); and c) Clinical culture (establish professional norms, values, roles, and expectations) • Model includes five key components: 1) Creation of partnership between health clinic and local community organizations 2) Recruit staff to receive in-depth IPV training and become healthcare advocates 3) Provide saturation training for all clinic staff members 4) Facilitate self-directed change in clinic systems, including the creation of clinical and administrative policies and procedures • Development of new clinic culture (e.g., new norms and values) as a result of completing components 1 through 4
*Primary Health Care Family Violence Responsiveness Evaluation Tool*	• Provides key system elements to guide effective response to family violence and quality improvement benchmarks • Encompasses 143 indicators within 10 categories organized to guide response development (e.g., from Governance & Leadership to Quality Improvement)
*General Practitioners’ Perceived Readiness to Identify and Respond to Intimate Partner Abuse Scale (GRIPS)*	• 30-item questionnaire with a four-point Likert scale (1 = *strongly disagree*, 2 = *disagree*, 3 = *agree*, 4 = *strongly agree*) • Informed by three sources: data generated by individual interviews with a purposive sample of general practitioners; perceived self-efficacy literature; and literature on general practitioners’ identification and responses to IPV victimization • Addresses six relevant dimensions: attitudinal readiness; personal views and values; emotional readiness; self-efficacy; knowledge of IPA issues; and communication skills

These models/tools informed the generation of key concepts that should be included in a family violence in PHC settings within Alberta tool, see Table 3. Concepts were extracted from each tool/model as they were identified by the authors who developed them. A key theme that emerged from the REA for enhancing PHC provider readiness is multidisciplinary teamwork and collaboration [104–107]. Readiness to address family violence is enhanced through having a supportive team environment and collaboration with family violence specialists (e.g., sexual assault nurse examiner, family violence advocate) who strengthen the PHC team’s self-efficacy and perceived preparation.

**Table 3:** Key concepts from identified tools/models

Name of Tool	Key Concepts Used in Delphi Process
General Practitioners’ Perceived Readiness to identify and respond to Intimate Partner Abuse Scale (GRIPS)	• Self-efficacy • Motivational readiness • Emotional readiness
Commitment/Advocacy/Trust/Collaboration/Health System (CATCH) Model	• Commitment • Advocacy • Trust • Teamwork • Health systems support
Health System Readiness Assessment Tool (HSRAT)	• Leadership • Governance • Financing • Infrastructure • Coordination • Values and attitudes • Service delivery • Information and documentation • Health workforce
Primary Health Care Family Violence Responsiveness Evaluation Tool	• Governance • Leadership • Collaboration • Policies and procedures • Education • Quality improvement • Resourcing • Documentation • Physical environment • Workplace culture • Routine inquiry/assessment
Health care Can Change from Within (HCCW) Model	• Partnership • Advocacy • Saturation • Change • Culture
Physician Readiness to Manage Intimate Partner Violence Survey (PREMIS)	• IPV knowledge • IPV training and background • Opinions (preparation, legal requirements, workplace issues, self-efficacy) • Practice issues

### Phase 2: Exploration of readiness to assess family violence in PHC settings within the Alberta context

Based on the exploration of concepts with panel members, an additional three concepts were added including: patient-centered care (e.g., trust, relationship building between provider and patient), cultural awareness and sensitivity (e.g., responding without prejudice and judgement to patient disclosures of family violence), and trauma-and-violence informed care (e.g., awareness of structural and system inequities, recognizing social and cultural realities of patients). Combined with concepts identified from existing tools/models, this resulted in a total of 16 concepts. The consensus among panelists to include ‘patient-centered care’ as a new concept in the tool was based on the view that patient-centered care extends beyond the role of patients as active participants in their own care (i.e., as suggested in the CATCH model), to PHC providers having a deep understanding of personal, social, cultural, and environmental factors that shape experience with family violence and access to healthcare. In other words, how the social determinants of health interact within the patient, their healthcare experience, and decision to disclose family violence.

### Phase 3: 3-round Delphi consensus-building process

A total of nine panelists agreed to participate in the Delphi consensus-building procedure including 8 females and 1 male who work in a variety of settings including a suburban primary care clinic (*n* = 2), an urban Indigenous-focused primary care clinic (*n* = 1), an urban hospital (*n* = 1), and community agencies (*n* = 4). Of the nine experts who participated, two identified as Indigenous. During round 1 voting, all concepts met the criteria to be included in the tool being developed (see Table 4).

**Table 4:** Findings from round 1 of Delphi consensus building process-concept voting

Concept and description	Median vote
Advocacy (e.g., champions to help with supporting systems change, having embedded advocates in the organization, advocating on behalf of patients)	9
Patient-centered care (e.g., trust, relationship building between provider and patient)	9
Teamwork and collaboration (e.g., multidisciplinary team models, composition of PHC team)	8.5
Health systems support and partnerships (e.g., having an enabling policy environment for health systems response to family violence)	9
Leadership and governance (e.g., having a strategic plan for family violence screening, use of practice facilitators)	9
Resources and infrastructure (e.g., dedicated budget for family violence programs and/or health promoting programs focused on the social determinants of health and violence prevention, embedded family violence advocates or social workers on site)	9
Physical environment (e.g., maximizing safety of the patient, having a safe and private environment for identification and assessment including posters related to violence and abuse for public display)	8.5
Values, attitudes, and workplace culture (e.g., individual and organizational commitment to family violence or addressing non-medical determinants of health)	8.5
Coordination and service delivery with community sector (e.g., collaboration with community agencies, integrated family violence service delivery model, referral pathways)	9
Information, documentation, and routine inquiry (e.g., prompts for providers within an electronic medical record system to inquire about factors that may contribute to violence, having a family violence screening tool, standardized family violence assessments)	9
Policies, procedures, legalities (e.g., mandatory training for clinicians and staff on trauma and violence informed care, reporting requirements of suspected or disclosed child abuse and violence)	9
Education and training (e.g., skill-based training on family violence, how to recognize the signs or risk factors for family violence)	8.5
Quality improvement and change (e.g., supporting culture of quality improvement, evaluating and assessing provider and staff confidence in identifying and responding to family violence)	8.5
Provider awareness of own beliefs, biases, and perceived knowledge of family violence (e.g., taking time to reflect individually and within the PHC team)	8
Cultural awareness and sensitivity (e.g., responding without prejudice and judgement to patient disclosures of family violence)	9
Trauma and violence informed care (e.g., awareness of structural and system inequities, recognizing social and cultural realities of patients)	9

After confirming that all concepts should be included in the proposed tool for PHC in Alberta, round 2 included voting on all indicators that were extracted from existing tools/models and adapted to reflect outputs from the exploratory meeting. In round 2, all indicators met the inclusion criteria to be included in the tool, see Table 5.

**Table 5:** Findings from round 2 of Delphi consensus building process-indicator voting

Indicator	Median vote
***Advocacy***	
Do you raise awareness about family violence and gender equity as an important issue for your practice?	8
Do you ask your patients about potential social challenges in a sensitive and culturally acceptable way, provide them with advice, refer them to local support services, and facilitate access to these services?	9
Do you use a clinical decision aid, practice guideline, or other tool within your everyday practice to assess structural vulnerabilities (negative health outcomes imposed by social determinants of health)?	7
Does the PHC practice/organization integrate patient social support navigators, family violence advocates, or social workers into the workplace?	9
Do you work with the regulated health professions and colleges to advocate for improving workforce capability and integrated, interdisciplinary, and sustainable supports for family violence within PHC?	8.5
***Patient-centered care***	
Do you create an environment where the patient feels safe enough to disclose their experiences and trusts you?	9
Are holistic assessments for health provided by a dedicated PHC team that is tailored to the patient’s various needs, and considering of their social circumstances?	9
Are procedures in place to ensure patient safety within the PHC practice?	8.5
Are trained interpreters available (including access to telephone interpreter service) for working with patients if English is not the first language?	9
***Teamwork and collaboration***	
Do you participate in a multidisciplinary team within your PHC practice?	9
Does your organization utilize communication strategies that promote intra-team communication, collaborative decision-making, and warm handoffs between team members?	8
Does your PHC organization demonstrate a team culture and interdisciplinary atmosphere of trust where contributions are valued?	8
Is there good understanding of roles within the PHC team and respect for autonomy?	8
Are patient social support navigators, family violence advocates, or social workers embedded within the PHC team?	9
Does the PHC practice collaborate with: (i) Community-based agencies, including domestic/family violence agencies or networks (ii) Local communities (iii) Community service providers (e.g., sexual assault centers, women’s shelter) (iv) Criminal justice sector (e.g., police) (v) Child protection services?	9
***Health systems support and partnerships***	
Does the PHC practice/organization encourage reflection and learning to build sustainable systems for meeting the health, safety, and recovery needs of women, children, and their families who are at-risk of, or currently experiencing family violence?	7.5
Is there strategic and continuous monitoring with feedback to ensure health service effectiveness and systems change?	7.5
***Leadership and governance***	
Is family violence included in the PHC organization’s (e.g., Primary Care Network (PCN)’s) strategic plan or directions?	8.5
Do PHC providers, staff, and clinic managers support addressing violence against women/family violence (for example, willing to provide care, supportive of sending staff to training)?	9
Are there confidential mechanisms to receive feedback from families about services, including any grievances or violations of rights in the health facility (for example, a helpline, ombudsperson, complaint box)?	7
Is there a workplace policy addressing discrimination and violence, including sexual harassment faced by PHC providers and staff themselves?	8
Does your PHC organization engage with Indigenous Elders and Knowledge Holders in the planning of care and support for Indigenous patients?	8
Resources and infrastructure	
Does the PHC practice/organization allocate adequate financial resources for family violence programs? (e.g., funding training, community activities, resources including a family violence coordinator or on-site family violence advocate)	7.5
Is there a space (for example, a room or area) available for private and confidential consultation? (e.g., space that ensures the patient cannot be seen or heard from outside)	9
Are trained interpreters available (including access to telephone interpreter service) for working with patients if English is not the first language?	8.5
Is appropriate information on healthy relationships and healthy conflict-resolution strategies and safety planning available to all patients, not only to those who are identified for intimate partner violence?	8
***Physical environment***	
Are there posters related to violence/abuse on public display in the waiting room?	8
Are brochures or other information resources on family violence, healthy relationships and healthy conflict resolution, and safety planning publicly available that include referral information for local services on: (i) Child abuse and neglect (ii) Partner abuse (iii) Elder abuse and neglect (iv) Sexual assault/abuse?	8.5
Are resources with referral information for relevant culturally specific services available within the PHC practice?	9
***Values, attitudes, and workplace culture***	
Does your PHC organizational culture demonstrate recognition of family violence and gender equity as important issues for the health service?	9
Do you have a personal commitment to family violence and gender equity?	8.5
Does the PHC practice/organization have a formal (written) assessment of staff ’s knowledge and attitudes about family violence, and their competence and comfort in assessing family violence?	8
Is there training on cultural and structural competency for PHC providers and staff, including orientation for new employees?	9
***Coordination and service delivery with community sector***	
Is there a referral system in place across different health services and between health and other sectors (for example, a referral directory/pathway) to provide information to survivors about available services?	9
Have other services (for example, police) and organizations (for example, local community-based agencies working on domestic/family violence) been informed about available health services?	8
***Information, documentation, and routine inquiry***	
Are there intake forms/registers and confidentiality mechanisms (for example, secure storage and removal of identifying information) for recording information about patient’s experience of violence and care received?	9
***Policies, procedures, legalities***	
Are there written protocols for the provision of health care to patients subjected to violence?	8
Do PHC providers, staff, and new employees receive training in cultural competency?	8.5
Does the PHC practice/organization have policies or procedures to address implicit biases to raise awareness of the potential harms of holding negative explicit attitudes towards some patients?	8
***Education and training***	
Are there mechanisms to provide ongoing mentoring, supervision, and support to PHC providers on how to recognize the signs of family violence?	9
Including yourself, how many providers at your practice have participated in an intimate partner violence training course (e.g., watched a video, attended a lecture or talk, attended a skills-based training or workshop, residency/fellowship/other post-graduate training)?	8.5
Does your PHC practice/organization use practice facilitators to support integration of education and learning into practice?	8.5
***Quality improvement and change***	
Are indicators and data to monitor the health response to violence against women/family violence being collected, compiled, and used to improve services?	7.5
***Provider awareness***	
Do you engage in self-reflexivity about your own biases or to identify core skills that can be learned in the development of culturally safe relationships with your patients?	7.5
Do you feel you have sufficient knowledge to assist individuals in addressing situations of family violence?	9
***Cultural awareness and sensitivity***	
Do you create an environment where the patient feels safe enough to disclose their experiences and trusts you?	9
Do policies and procedures support cultural safety: (i) Is assessment and inquiry specifically recommended in family violence regardless of the patient’s cultural background? (ii) Do PHC providers and staff participate in cultural safety training (e.g., Indigenous Health Cultural Competency curriculum)?	7.5
***Trauma and violence informed care***	
Have you and other providers within your PHC practice completed trauma-informed training?	9

Based on written feedback received during round 2, eight indicators were reworded, and two additional indicators were added to be voted on in the final round (see Table 6).

**Table 6:** Changes made to indicators based on feedback from round 2

Indicator in round 2	Changes made to indicator
Do you raise awareness about family violence and gender equity as an important issue for your practice?	Do you raise awareness about gender equity and take appropriate measures to address gender bias as well as other forms of discrimination as important issues for your practice?
Do you create an environment where the patient feels safe enough to disclose their experiences and trusts you?	Do you create a culturally welcoming and inclusive environment where the patient feels safe enough to share personal experiences within their home environment or intimate relationships and trusts you?
Are procedures in place to ensure patient safety within the PHC practice?	Are procedures in place to report and respond to safeguarding concerns among children and youth at risk of violence and abuse?
Does the PHC practice collaborate with: (i) Community-based agencies, including domestic/family violence agencies or networks (ii) Local communities (iii) Community service providers (e.g., sexual assault centers, women’s shelter) (iv) Criminal justice sector (e.g., police) (v) Child protection services?	Does the PHC practice collaborate with: (i) Community-based agencies, including domestic/family violence agencies or networks (ii) Local communities (iii) Community service providers (e.g., sexual assault centers, women’s shelters, and multicultural service agencies) (iv) Criminal justice sector (e.g., police) (v) Child protection services (vi) Child advocacy centers
Are there posters related to violence/abuse on public display in the waiting room?	Are there visual images and posters in multiple languages related to healthy relationships, healthy parenting, and recognizing the signs of family violence on public display in the waiting area and examination room?
Is there training on cultural and structural competency for PHC providers and staff, including orientation for new employees?	Is there training on cultural and structural competency for PHC providers and staff, including orientation for new employees and re-fresher training for current staff?
Is there a referral system in place across different health services and between health and other sectors (for example, a referral directory/pathway) to provide information to survivors about available services?	Is there a referral system in place between the PHC service and community-based sector services (for example, a referral directory/pathway) to provide information to survivors about available services?
Do you feel you have sufficient knowledge to assist individuals in addressing situations of family violence?	Do you feel you have sufficient knowledge of cultural practices, protocols, beliefs related to family violence to assist patients from different cultural backgrounds?
***New item***	Does the practice have knowledge of local Indigenous and cultural-ethnic groups, protocols for communicating with groups including Indigenous peoples and persons of immigrant and refugee backgrounds, and have a strategy for active engagement for local, culturally diverse groups?
***New item***	Does your PHC practice/organization promote the use of a tool to support providers to work in a trauma- and violence-informed way?

In round 3, panelists voted on the indicators for the final time for inclusion in the proposed tool. In the final voting, all indicators met the inclusion criteria. This resulted in a total of 60 items being held for inclusion in the readiness to assess family violence in PHC within Alberta tool.

## Discussion

There is an increasingly urgent demand to establish family violence intervention programs focused on identification, assessment, and prevention within PHC, necessitating a more thorough examination of the preparedness of healthcare providers and organizations to integrate a comprehensive response to family violence. PHC is considered a priority setting where a disproportionate number of people impacted by family violence may present [108]. Given the consecutive rise in family violence rates over the years in Canada [109], it is imperative to promptly identify any gaps in policy and practice that hinder PHC organization’s capacity to effectively respond to this pressing issue. The present work contributes a conceptual framework with indicators that may be employed to assess a PHC setting’s readiness to address family violence and guide implementation of comprehensive family violence intervention programs, described as a KTA process. The purpose of this endeavor is not to provide a fully developed tool suitable for immediate utilization by PHC organizations, rather, we present the preliminary findings as an initial step towards validating this tool for practical implementation.

Importantly, this work has identified several crucial areas that are currently absent in existing tools/models to measure readiness, particularly in terms of considerations for delivering care to Indigenous patients. Limited information exists on what factors enable the implementation of family violence tools/models that are inclusive of the social and cultural realities of Indigenous peoples. The issue of family violence within Indigenous communities is a pressing concern that necessitates careful attention and culturally sensitive approaches to address the unique challenges faced by Indigenous peoples [110, 111]. Recognizing and addressing historical factors, particularly the impact of colonization and colonial structures, is of utmost importance in understanding and addressing the enduring issue of family violence among Indigenous peoples. By prioritizing the inclusion of Indigenous perspectives and cultural safety within our tool, we have the opportunity to redefine knowledge and power dynamics at both the healthcare provider and organizational levels.

Our work has also emphasized the importance of including children and youth in comprehensive safeguarding practices. Children and youth are often overlooked or neglected in the screening of family violence within healthcare settings, highlighting the urgent need for improved protocols and interventions to ensure their safety and well-being. Our tool serves as an impetus for PHC practices to establish sustainable systems that effectively address the health and safety requirements of women, men, children, youth, and their families who are at risk of experiencing family violence and abuse. In alignment with a comprehensive approach to family violence, our tool strongly advises PHC organizations to enhance their capacity to respond to diverse forms of family violence across all population groups, promoting an inclusive and encompassing approach.

While the final panel ratings successfully met the inclusion threshold for all statements during the Delphi consensus-building process, certain points of discussion were identified. For instance, several panelists emphasized the importance of extending safety plans beyond patients to also encompass PHC providers and staff at risk of or experiencing violence in their own relationships. Another point of discussion centered around the need to ensure inclusivity for all equity-deserving groups by establishing protocols for communication and engagement with Indigenous peoples, individuals from immigrant and refugee backgrounds, and members of the LGBTQIA2S + community. Finally, numerous panel members strongly advocated for an effective and comprehensive systems response to family violence in PHC, emphasizing the importance of collaboration with community agencies and the broader healthcare system. As a result, they introduced an additional indicator that evaluates whether the PHC organization actively promotes integrated health services delivery with a focus on coordinated care across health, social and community sectors.

While the process of developing the readiness to implement family violence programs tool in PHC settings was conducted with rigor and transparency, it is important to acknowledge and emphasize the study’s limitations. First and foremost, the utilization of a REA instead of a systematic review may have led to the potential oversight of certain evidence [78]. Moreover, the outcomes of the REA underscored the limited research available in this specific domain, necessitating the inclusion of numerous concepts based on expert opinions within this study. Another notable limitation is that, although our Delphi panel of experts identified gaps, particularly regarding areas that are sensitive to Indigenous peoples and communities, we did not directly involve or incorporate the lived experiences of Indigenous peoples affected by family violence in our conceptualization of readiness. It is important to recognize that a different panel composition might have yielded distinct concepts. Our panel, being representative of the Alberta health system context, implies that our findings should be considered within this context. Another limitation is the small size of the sample on the panel (*n* = 9), which may have resulted in all indicators being rated highly and therefore minimal reduction. A larger panel size would have also increased the reliability of group judgements. Furthermore, of the experts on this panel, there was only one primary care family physician, which is a notable limitation as the perspective and experience of family physicians differ from other healthcare professionals such as nurses as well as family violence specialists. As PHC interactions are centered around the family physician-patient relationship in Alberta, it is important that family violence interventions shape how they understand and perform their roles in caring for affected patients. Yet, as stated earlier a focus of our study was to provide guidance on assessing readiness for family violence response among the entire inter-professional PHC team. Lastly, despite the final panel ratings meeting the inclusion threshold for all indicators during the Delphi consensus-building process, this led to a lengthy assessment where certain items were found to be repetitive across different concepts. In future advancements of this work, pilot testing will be conducted to gather data that can be utilized to streamline and reduce the number of indicators.

Future research will work on streamlining and refining the tool by strategically reducing the number of items in the tool. The 16 concepts presented are qualitative categories with inherent nuances, and further exploration of items that apply across concepts through co-occurrences may lead to a more concise and robust scale for use. Furthermore, a deeper investigation into the response options (i.e. yes/no versus Likert scales) may enhance the sensitivity of the assessment tool. To ensure the applicability of the tool, PHC experts and stakeholders need to be further engaged and their input sought for refining both the concepts and items. Finally, pilot data from various sites will enable robust statistical analyses to improve the tool’s applicability in diverse PHC contexts.

## Conclusion

In conclusion, the work carried out in this study serves as a foundation for future research and practical advancements in family violence intervention programs. By identifying the components that foster readiness for implementing comprehensive family violence programs, we aim to assist PHC organizations in reorienting themselves to effectively address an escalating public health crisis. The presented work will be further developed to create a validated tool that can be widely utilized within PHC settings.

## Data Availability

The datasets generated and/or analysed during the current project are not publicly available due to privacy and ethical restrictions but are available from the corresponding author on reasonable request.

## Abbreviations

IPV: Intimate partner violence
PHC: Primary health care
PCN: Primary Care Network
DVA: Domestic violence and abuse
KTA: Knowledge to Action
REA: rapid evidence assessment
